# Prevalence and outcomes of Urinary tract infections caused by Enterobacterales resistant to third-generation cephalosporins in the Emergency Department: results from UTILY cohort, a prospective multicentre study

**DOI:** 10.1007/s15010-025-02547-3

**Published:** 2025-05-09

**Authors:** Caterina Monari, Lorenzo Onorato, Alessandro Cornelli, Margherita Macera, Enrico Allegorico, Andrea Ferraro, Carmine Nasta, Maria Teresa Florio, Kim Russo, Piero Bianco, Vita Dora Iula, Fabio Giuliano Numis, Giovanna Guiotto, Mauro Giordano, Rosa Raucci, Ferdinando Dello Vicario, Rodolfo Nasti, Evaluna Perez Guillen, Nicola Coppola, for UTILY study group*, Lorenzo Onorato, Margherita Macera, Caterina Monari, Federica Ciminelli, Ilaria De Luca, Annabella Salvati, Alessandro Cornelli, Nicola Coppola, Fabio Giuliano Numis, Enrico Allegorico, Piero Bianco, Stefano Aiello, Stefano Viola, Maria Rocco, Biagio Migliaccio, Antonio Augiero, Nicola Crispino, Fabio Mari, Dalila Guesmi, Giuseppe Pomilla, Iacopo Vespoli, Andrea Ferraro, Vita Dora Iula, Giovanna Guiotto, Carmine Nasta, Angela Di Sisto, Federico Schettini, Vincenzo Brunelli, Romeo Morelli, Francesca Palumbo, Alfredo Palumbo, Antonia Ida Facciuto, Valeria Palo, Martina Finelli, Antonio Allegretto, Mariachiara Giordano, Mauro Giordano, Maria Teresa Florio, Anna Amato, Anna Santagata, Adelaide Mariniello, Lucrezia Carozza, Nicola Quaranta, Vincenza Serrao, Augusto Delle Femmine, Ilaria Guida, Annalisa Amelia, Federica Miglietta, Rosa Raucci, Roberta Sciorio, Kim Russo, Federica Esposito, Filomena Fabozzi, Luca De Capua, Gennaro Borrelli, Rodolfo Nasti, Evaluna Perez Guillen, Antonio Voza

**Affiliations:** 1 Department of Mental Health and Public Medicine, Section of Infectious Diseases, University of Campania Luigi Vanvitelli, Via L. Armanni 5, 80131 Naples, Italy; 2 UOC Medicina d’Emergenza ed Urgenza, Ospedale Santa Maria delle Grazie, ASL Napoli 2 Nord, Pozzuoli, NA Italy; 3 Medicina di Emergenza-Urgenza, Ospedale del Mare, ASL Napoli 1 Centro, Naples, Italy; 4 Department of Advanced Medical and Surgical Sciences; Emergency Department, University of Campania Luigi Vanvitelli, Marcianise (Caserta), Italy; 5 Pronto Soccorso, P.O. Moscati, 81031 Aversa, Caserta Italy; 6 UOSD Pronto Soccorso-OBI, Ospedale San Giuliano, Asl Napoli 2 Nord, Giugliano, NA Italy; 7 Pronto Soccorso, Ospedale Betania, Naples, Italy; 8 Department of Biomedical Sciences, IRCCS Humanitas Research Hospital, Milan, Italy

**Keywords:** ESBL, 3-Generation cephalosporin resistant microrganisms, Enterobacterales, *E. coli*, *K. pneumoniae*, UTI, Emergency department

## Abstract

**Introduction:**

In accordance with the spread of drug-resistant bacteria worldwide, an increase in the prevalence of Antimicrobial Resistance (AMR) among pathogens causing urinary tract infections (UTIs) has been described globally. The aim of this study was to describe the prevalence and outcome of UTIs caused by third-generation cephalosporin-resistant (3GC-R) *Enterobacterales* in a prospective cohort of patients admitted to Emergency Department (ED).

**Materials and methods:**

We conducted an observational prospective multicentre study, involving 7 healthcare facilities, enrolling all consecutive adult patients admitted to ED with a microbiologically confirmed diagnosis of UTIs caused by *Enterobacterales*. The primary outcomes were the prevalence of UTIs caused by 3GC-R *Enterobacterales*, and 30-day mortality.

**Results:**

During the study period, we included 288 patients with urinary tract infection: 41.7% of subjects were males, median age was 72 years (IQR 56–81). The most frequently isolated pathogen was *Escherichia coli* (70.5%); 35.9% of all pathogens isolated were non-susceptible to 3GC. At multivariate logistic regression analysis, admission to a hospital (OR 3.31, 95% CI 1.41–7.75, *p* = 0.006) or a long-term care facility (OR 4.87, 95% CI 1.16–20.36, *p* = 0.03) in the previous three months was independently associated with isolation of a 3GC-R pathogen. Regarding the clinical outcomes, 22 out of 217 (10.1%) patients completing follow-up died at 30 days. At multivariate analysis 7-day clinical response was the only variable associated with 30-day mortality (OR 0.11, 95% CI 0.04–0.36, *p* < 0.001).

**Conclusions:**

In our study, 35.9% of pathogens isolated in urine cultures of patients with community-acquired UTIs were non-susceptible to 3GC. In the ED, the knowledge of local epidemiology and of risk factors for antimicrobial resistance is of paramount importance for choosing the right empiric therapy and setting up local guidelines.

**Supplementary Information:**

The online version contains supplementary material available at 10.1007/s15010-025-02547-3.

## Introduction

Urinary tract infections (UTI) are among the most common infections in both community and hospital settings and are characterised by high morbidity rates, a decreased quality of life, and significant economic costs secondary to treatment and hospitalisation [1, 2]. UTIs are also one of the leading causes of access to emergency departments (ED), with urosepsis accounting for 5–7% of severe sepsis cases [3].

Globally, 404,61 million cases of UTIs and 236,790 related deaths have been reported in 2019 with an 60.4% increase in cases and an increasing age-standardized mortality rate from 2.77 to 3.13/100,000 during the period of 1990–2019 [2].

The most frequently isolated pathogen is uropathogenic *Escherichia coli*, which is responsible for approximately 85% of all UTIs, since it belongs to the intestinal bacterial flora and thus easily colonises the urinary tract [1]. Other *Enterobacterales*, including *Klebsiella* spp., *Proteus* spp., *Morganella morganii*, *Providencia* spp., and *Serratia* spp. are more frequently isolated in nosocomial forms.

The phenomenon of Antimicrobial Resistance (AMR), including resistance to third-generation cephalosporins (3GC), poses a major threat to Global Health worldwide, with a significant negative impact on therapeutic options and mortality [4]. For this reason, in 2024 the World Health Organization (WHO) published a list of antibiotic-resistant “priority pathogens” to guide and promote research and development of new antibiotics, and indicated 3GC-resistant (3GC-R) *Enterobacterales* as “critical”, along with carbapenem-resistant *Enterobacterales* and carbapenem-resistant *Acinetobacter baumannii* [5].

An estimated 1,27 million deaths were directly attributable to antimicrobial-resistant bacteria in 2019, with a further estimated 4,95 million associated deaths [4]. Moreover, in the same year, an increase in the prevalence of AMR among pathogens causing UTIs was described, with 64,9 thousand deaths attributed and 0,26 million deaths associated with bacterial AMR: among them, 3GC-resistant (3GC-R) and fluoroquinolones-resistant *E. coli* were the leading cause of death [6].

The last EARS-net record [7] reported that in Europe 53.2% of the E. coli and 38.4% of the *K. pneumoniae* isolates were resistant to at least one of the antibiotics under surveillance in 2021, *i.e.*, aminopenicillins, fluoroquinolones, 3GC, aminoglycosides and carbapenems; of these, 14.3% of *E. coli* and 34.3% of *K. pneumoniae* were resistant to third-generation cephalosporins. However, the epidemiology of drug-resistant bacteria shows a great variability across Europe [4]. Italy is among the countries with the highest AMR prevalence in *Enterobacterales*, with 23.8% of *E. coli* and 53.3% of *K. pneumoniae* resistant to 3GC [7]. The knowledge of local epidemiology is essential to set up effective empiric antibiotic therapy regimes, especially in settings such as the emergency departments, where the decision-making process is rapid, and the pursuit of a microbiological result is much more challenging.

The aims of this study were to describe the prevalence and outcome of UTIs caused by 3GC-R *Enterobacterales*, and to assess the factors associated with 3GC-R and 30-day mortality.

## Materials and methods

### Study design and setting

We performed an observational prospective multicenter study, involving six EDs in the Campania Region in southern Italy, and 1 ED in Lombardy, northern Italy, from February 2023 to July 2024. These seven centers, coordinated by the Infectious Diseases unit, have cooperated in several clinical investigations participating in the UTi in ItaLy (UTILY) cohort and use the same clinical approach and laboratory methods.

### Inclusion and exclusion criteria

All consecutive adult patients with a microbiologically confirmed diagnosis of community-acquired UTIs, *i.e.*, cystitis, pyelonephritis, renal or perinephric abscesses, catheter-associated urinary tract infections, urosepsis or septic shock secondary to UTI, caused by *Enterobacterales* were enrolled.

Patients below 18 years of age, those with negative urine culture or those who refused to provide an informed consent were excluded from the study.

### Data collection

For the patients included in the study, we collected the following information in a pre-defined case report form (CRF): age, sex, comorbidities, Charlson Comorbidity Index (CCI), hospitalization or antimicrobial therapies in the previous 90 days, invasive procedures in the previous 30 days, presence of urinary devices (urinary catheter, ureteral stent, nephrostomy), any ongoing antibiotic treatment at the time of UTI diagnosis, severity of infection, SOFA score, empiric and targeted antibiotic therapy, hospitalization need, isolated pathogens and antimicrobial susceptibility profile, duration of treatment, length of stay (LoS), clinical response at 7 days, and mortality at 7 and 30 days.

### Definitions and outcomes

The diagnosis of UTI was made according to the CDC/NHSN Surveillance Definitions for Urinary Tract Infection Events [8] and to the recent guidelines of the European Association of Urology [9]. We defined a urine culture as positive when detecting a potential pathogen at a high number of colony forming units (CFU), *i.e.*, ≥ 10^5^ (CFU)/mL. Patients with cultures detecting mixed flora, non-speciated *Streptococcus* or *Staphylococcus* organisms, or *Candida* species were excluded from the analysis. The diagnosis of sepsis or septic-shock was achieved according to the SEPSIS-3 definitions [10]. Pathogens were defined non-susceptible to 3GC if they showed resistance or intermediate susceptibility in vitro to ceftazidime and/or cefotaxime and/or ceftriaxone. Lastly, empiric treatment was defined appropriate based on in vitro susceptibility against the isolated strain.

The primary outcomes of our study were the prevalence of community-acquired UTIs caused by 3GC-resistant *Enterobacterales*, and the 30-day mortality. Secondary outcomes included the 7-day clinical response, defined as the resolution of signs and symptoms of infection at 7 days after the initiation of treatment, and the 7-day mortality.

### Statistical analysis

Continuous variables were reported either as mean and standard deviation (SD) if normally distributed, or as median and interquartile range (IQR) if not normally distributed. Categorical variables were expressed as absolute numbers or relative frequencies. Comparison between groups were performed using the Pearson chi-squared test for categorical variables, using exact procedures if needed, and the Student’s t-test or Mann-Whtiney U tests for continuous variables. Differences were considered statistically significant at *p* < 0.05 (two-tailed tests). Variables presenting a *p* value < 0.05 at univariate analysis that could have a causal correlation with the mortality outcome on the basis of the evidence available in scientific literature were included in a binary logistic regression model, to identify predictors of 3GC-resistance and 30-day mortality; variables with a prevalence < 5% in the population were excluded from the model. A power analysis was conducted to support the logistic regression model for the analysis evaluating the predictors of 3GC-resistance. For this analysis, considering a risk of 20% among patients without previous hospital admission, and 45% probability in patients presenting the risk factors, as reported in a previous study conducted on the same cohort [11], with an alpha of 0.05, we estimated a power > 0.9 with our sample size. No power analysis was conducted for the analysis of predictors of mortality. Analyses were performed by SPSS 23.0 (IBM, Armonk, NY, USA).

### Ethics approval

The study was approved by the Ethic Committee of the University of Campania Luigi Vanvitelli, Naples (protocol n◦ 35093/2023). All methods used in this study were in accordance with the ethical standards of the institutional and/or national research committee and the Helsinki Declaration of 1975, revised in 1983. Informed consent was obtained from all participants included.

## Results

### Epidemiological, clinical and microbiological characteristics of enrolled patients

**Fig. 1:**
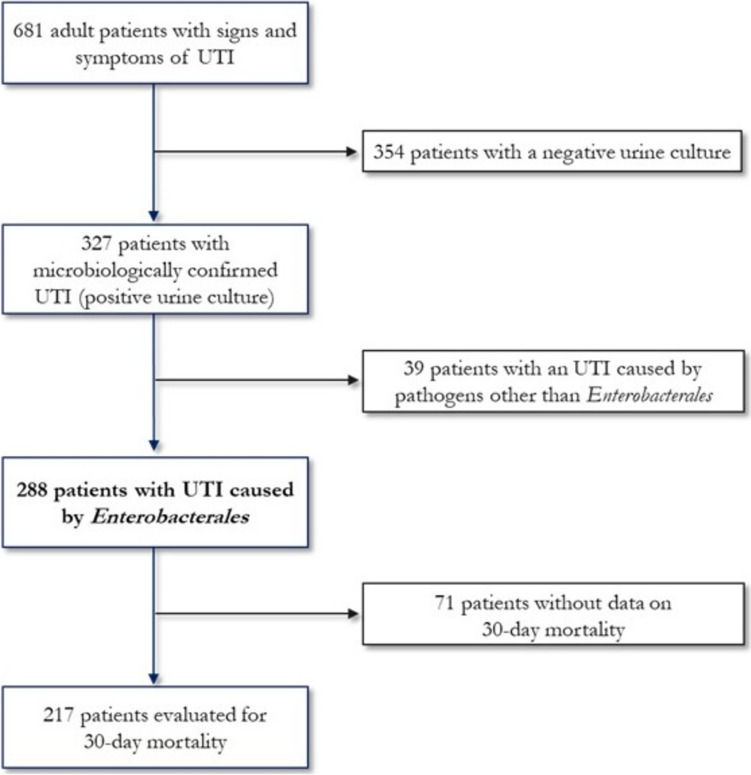
Flowchart of patients selection process

During the study period, 681 patients were admitted to one of the 7 EDs participating in the study with signs and symptoms consistent with UTI; 327 of them presented a positive urine culture and 288 a positivity for *Enterobacterales*. Figure 1 illustrates the flowchart of the selection of patients included in the study. Total 288 patients were enrolled in the present study: 41.7% of subjects were males, median age was 72 years (interquartile range, IQR, 56–81), and median Charlson Comorbidity Index (CCI) 1 (IQR 0–4). Demographic, clinical and microbiological characteristics of patients are described in Table 1. The most frequent comorbidity was diabetes mellitus (27.4%), followed by chronic kidney disease (22.6%). Among risk factors, the most frequent was the presence of a urinary catheter (17.4%), while 37 patients (12.8%) underwent invasive urinary procedures in the previous 30 days. None of the patients enrolled had known rectal colonisation by MDR germs, 22.1% took antibiotics in the previous three months, 12.4% were admitted to a hospital and 3.7% to a long-term care facility in the previous three months.

**Table 1:** Demographic, clinical and microbiological characteristics of patients included in the study

Demographic variables	N = 288
Male, n (%)	120 (41.7%)
Age, median years (IQR)	72 (56–81)
Charlson comorbidity index, median (IQR)	1 (0–4)
*Comorbidities and risk factors*	
Comorbidities, n (%):	
Diabetes mellitus	79 (27.4)
Heart failure	44 (15.3)
Chronic kidney disease	65 (22.6)
Haemodialysis	8 (2.8)
COPD	47 (16.3)
Solid cancer	40 (13.9)
Haematological cancer	11 (3.8)
Neutropenia	2 (0.7)
Chronic hepatopathy	19 (6.6)
Urinary calculosis	34 (11.8)
Solid organ transplantation	2 (0.7)
Benign prostatic hypertrophy	32 (11.1)
Devices, n (%):	
Urinary catheter	50 (17.4)
Nephrostomy	7 (2.4)
Ureteral stent	22 (7.6)
Invasive procedures on the urinary tract in the previous 30 days, n (%)	
Surgery	4 (1.4)
Endoscopy	7 (2.4)
Change of urinary catheter/nephrostomy/stent	26 (9.0)
None	251 (87.2)
Known rectal colonisation by MDR pathogen, n (%):	0 (0)
Antibiotic therapy in previous 3 months, n (%):	61/276 (22.1)
Current antibiotic therapy, n (%):	33/272 (12.1)
Hospitalisation in the previous 3 months, n (%):	33/267 (12.4)
Hospitalisation in Long-term facilities in the previous 3 months, n (%):	10/267 (3.7)
*Clinical and microbiological variables*	
Severity of infection, n (%):	
Non-sepsis	128/285 (44.9)
Sepsis	125/285 (43.9)
Septic shock	32/285 (11.2)
SOFA score, median (IQR)	1 (0–3)
Positive urine cultures, n (%):	288 (100)
Agent, n (%):	
*E. coli*	203 (70.5)
*Klebsiella* spp.	53 (18.4)
*Proteus* spp.	16 (5.5)
*Enterobacter* spp.	11 (3.8)
Other enterobacterales	4 (1.4)
3CG non-susceptible pathogens, n (%)	99/276 (35.9)
Empirical antibiotic therapy, n (%)	
Amoxicillin/clavulanate or ampicillin/sulbactam	120 (41.6)
Piperacillin/tazobactam	77 (26.7)
3rd generation cephalosporins	14 (4.9)
Carbapenems	6 (2.1)
Fluoroquinolones	16 (5.6)
Aminoglycoside	18 (6.3)
Trimetoprim/sulfamethoxazole	12 (4.2)
Iv Fosfomycin	7 (2.4)
Glycopeptides	4 (1.4)
Other	2 (0.7)
Unknown	28 (9.7)
Empirical combination therapy, n (%):	25 (8.7)
Empirical antibiotic therapy, n (%):	
Adequate	176 (61.1)
Not adequate	82 (28.5)
Unknown	30 (10.4)
Hospitalisation, n (%)	68/128 (53.1)
*Outcome*	
Clinical response at 7 days, n (%):	181/222 (81.5)
Mortality, n (%)	
At 7 days	12/223 (5.4)
At 30 days	22/217 (10.1)

The most frequently isolated pathogen was *Escherichia coli* (70.5%), while *Klebsiella pneumoniae* was identified in 53 (18.4%) patients. The majority of the patients enrolled showed severe disease, sepsis in 43.9% and septic shock in 11.2% of cases. Empiric antibiotic therapy, given to 8.7% of patients, was adequate in 61.1% of cases. The 30-day mortality was 10.1% (Table 1).

Supplementary Table 1 shows demographic, clinical and microbiological characteristics of patients, according to urban areas of origin. We evaluated the urban areas of Naples and Caserta, since they represented 95% of population included in the study.

### Antibiotic susceptibility patterns/profiles of isolated strains

Of the 288 patients enrolled, 35.9% harboured *Enterobacterales* non-susceptible (“R” or “I”) to 3GC; among them, 6 strains were “I” and 93 were “R”. Supplementary Table 2 describes the susceptibility profile of pathogens most frequently isolated in our cohort, *i.e., Escherichia coli, Klebsiella* spp., and other *Enterobacterales*.

*E. coli* (N = 203 strains) was non-susceptible to aminopenicillins in 47.2% of cases, to fluoroquinolones (ciprofloxacin) in 43.3%, to trimethoprim/sulfamethoxazole (TMP/SMX) in 34.1%, and to 3GC in about 30% (cefotaxime/ceftriaxone 30.2%, ceftazidime 31.1%). *Klebsiella* spp. (N = 53) was non-susceptible to aminopenicillins in 61.2% of cases, to piperacillin/tazobactam (P/T) in 55.1%, to fluoroquinolones (ciprofloxacin) in 55.1%, and to 3GC in about 48% (cefotaxime/ceftriaxone 49%, ceftazidime 46.9%); regarding aminoglycosides, *Klebsiella* spp. was non-susceptible to gentamicin in a quarter of cases (26.5%) but resistance to amikacin was never described (0%). Other *Enterobacterales* (N = 32)*,* encompassing *Proteus* spp., *Morganella morganii, Providencia* spp*. and Serratia* spp., were frequently non-susceptible to aminopenicillins (45.2%), fluoroquinolones (38.7%) and 3GC (61.3% ceftazidime and 22.6% cefotaxime/ceftriaxone), but lower rate of resistance to P/T (6.3%) was identified.

Supplementary Table 1 describes the distribution of isolates and their susceptibility profiles according to the urban areas of origin.

### Factors associated with the isolation of 3GC “non-susceptible” pathogens

In the Table 2 we compared the characteristics of 99 patients with UTI caused by a 3GC “non-susceptible” pathogen with those of 177 patients with UTI caused by a 3GC-susceptible (3GC-S) strain. We have evaluated 276 out of the 288 total patients because for 12 subjects the susceptibility profile of isolated strains was not available.

**Table 2:** Patient characteristics stratified according to isolation of 3GC-R *Enterobacterales*

	3GC-R	3GC-S	p value
N of patients = 276	99	177	
*Demographic variables*			
Male, n (%)	47 (47.5)	69 (38.9)	0.17
Age, median years (IQR)	73 (60–82)	70 (52–80)	**0.03**
Charlson comorbidity index, median (IQR)	2 (0–4)	1 (0–3)	**0.03**
*Comorbidities and risk factors*			
Comorbidities, n (%):			
Diabetes mellitus	27 (27.3)	49 (27.7)	0.94
Heart failure	14 (14.1)	26 (14.7)	0.90
Chronic kidney disease	25 (25.2)	39 (22.0)	0.54
Haemodialysis	3 (3.0)	5 (2.8)	0.92
COPD	10 (10.1)	35 (19.7)	**0.04**
Solid cancer	19 (19.2)	20 (11.3)	0.07
Haematological cancer	5 (5.0)	6 (3.4)	0.50
Neutropenia	1 (1.0)	1 (0.6)	0.68
Chronic hepatopathy	4 (4.0)	15 (8.5)	0.16
Urinary calculosis	13 (13.1)	20 (11.3)	0.65
Solid organ transplantation	2 (2.0)	0 (0)	0.06
Benign prostatic hypertrophy	8 (8.1)	22 (12.4)	0.27
Devices, n (%):			
Urinary catheter	21 (21.2)	28 (15.8)	0.26
Nephrostomy	5 (5.1)	2 (1.1)	**0.047**
Ureteral stent	11 (11.1)	11 (6.2)	0.15
Invasive procedures on the urinary tract in the previous 30 days, n (%)			
Surgery	2 (2.0)	2 (1.1)	0.56
Endoscopy	1 (1.0)	6 (3.4)	0.23
Change of urinary catheter/nephrostomy/stent	15 (15.2)	11 (6.2)	0.02
Antibiotic therapy in previous 3 months, n (%):	27 (27.3)	34 (19.2)	0.11
Current antibiotic therapy, n (%):	15 (15.2)	17 (9.6)	0.12
Hospitalisation in the previous 3 months, n (%):	21 (21.2)	11 (6.2)	** < 0.001**
Hospitalisation in long-term facilities in the previous 3 months, n (%):	7 (7.1)	3 (1.7)	**0.03**
*Clinical and microbiological variables*			
Severity of infection, n (%):			**0.003**
Non-sepsis	35/99 (35.4)	90/174(51.7)	
Sepsis	46/99 (46.5)	72/174(41.4)	
Septic shock	18/99 (18.2)	12/174 (6.9)	
SOFA score, median (IQR)	2 (0–4)	1 (0–3)	0.008
Agent, n (%):			**0.03**
*E. coli*	63 (63.6)	132 (74.6)	
*Klebsiella* spp.	26 (26.3)	24 (13.6)	
*Proteus* spp.	2 (2.0)	14 (7.9)	
*Enterobacter* spp.	5 (5.1)	6 (3.3)	
Other enterobacterales	3 (3.0)	1 (0.6)	
Empirical antibiotic therapy, n (%)			
Amoxicillin/clavulanate or ampicillin/sulbactam	33 (33.3)	85(48.0)	**0.02**
Piperacillin/tazobactam	44 (44.4)	33 (18.6)	** < 0.001**
3rd generation cephalosporins	1 (1.0)	10 (5.6)	0.059
Carbapenems	5 (5.1)	1 (0.6)	**0.01**
Fluoroquinolones	6 (6.1)	10 (5.6)	0.89
Aminoglycoside	5 (5.1)	13 (7.3)	0.46
Trimetoprim/sulfamethoxazole	3 (3.0)	8 (4.6)	0.54
Iv Fosfomycin	0 (0)	7 (3.95)	**0.045**
Glycopeptides	2 (2.0)	2 (1.1)	0.55
Empirical combination therapy, n (%):	9 (9.1)	15 (7.7)	0.86
Appropriate empirical antibiotic therapy, n (%):	43/91 (47.3)	125/157 (79.6)	** < 0.001**
Hospitalisation, n (%):	28/47 (59.6)	40/81 (49.4)	0.26
*Outcome*			
Clinical response at 7 days, n (%):	58/82 (70.7)	118/135 (87.4)	**0.002**
Mortality, n (%)			
At 7 days	7/82 (8.5)	5/136 (3.7)	0.13
At 30 days	13/79 (16.5)	9/133 (6.7)	**0.025**

Patients in the 3GC “non-susceptible” group were older (73 *versus,vs*., 70 years, *p* = 0.03), with more comorbidities (median CCI 2 vs. 1, *p* = 0.03), they more frequently had a nephrostomy (5.1% vs. 1.1%, *p* = 0.047), and underwent more frequent urinary device (catheter, nephrostomy or urinary stent) replacement in the previous 30 days (15.2% vs. 6.2%, *p* = 0.02). We found no significant differences in the antibiotic intake in the previous 3 months (*p* = 0.107), but we did find a significant difference in history of previous hospitalisation (21.2% vs. 6.2%, *p* < 0.001) and long-term care facility stay (7.1% vs. 1.7%, *p* = 0.03).

At multivariate logistic regression analysis, shown in Table 3, the admission to a hospital in the previous three months, was independently associated with isolation of a 3GC- “non-susceptible” pathogen, with an Odds Ratio (OR) of 3.04 (95% Confidence Interval, CI, 1.35–6.85, *p* = 0.007).

**Table 3:** Multivariate analysis for isolation of 3GC-R *Enterobacterales*

	OR	95% CI		*p* value
		Lower	Upper	
Age > 70 years	1.13	0.65	1.96	0.67
Charlson comorbidity index > 2	1.39	0.79	2.46	0.25
Change of urinary catheter/nephrostomy/stent	1.58	0.64	3.93	0.32
Hospitalisation in the previous 3 months	3.04	1.35	6.85	**0.007**

### Clinical characteristics at baseline and outcome according to the presence of 3GC “non-susceptible” pathogens

At baseline, patients with UTIs caused by 3GC “non-susceptible” strains experienced more severe infections (*p* = 0.003), with a higher SOFA score (2 vs. 1, *p* = 0.008) (Table 2). A different distribution of pathogens between the two groups also emerged, with *Klebsiella* spp*.* isolated more frequently in the study group (*p* = 0.003) (Table 2). Regarding empiric treatment, patients in the study group more frequently received piperacillin/tazobactam (44.4% vs. 18.6%, *p* < 0.001) and carbapenems (5.1% vs. 0.6%, *p* = 0.01), whereas aminopenicillins were the most prescribed drugs in the control group (48.0%, *p* = 0.018) (Table 2). Empiric therapy was less frequently adequate in the study group compared to the control one (47.3% vs. 79.6%, *p* < 0.001) (Table 2).

A significant worse 7-day clinical response (70.7% vs. 87.4%, *p* = 0.002) and a significant higher 30-day mortality (16.7% vs. 6.7%, *p* = 0.025) were observed in patients with infections caused by 3GC “non-susceptible” compared to 3GC-S pathogens (Table 2).

### Factors associated with 30-day mortality

Lastly, we compared 22 patients who died at 30 days with 195 who survived (Table 4). Patients who died presented a higher median age (79 vs. 72, *p* = 0.004), more frequently had a urinary catheter (*p* = 0.03), and were more frequently admitted to a long-term facility (*p* = 0.004). Moreover, they experienced more severe infections (*p* = 0.003), with a higher median SOFA score (4 vs. 1, *p* < 0.001), had a higher rate of UTIs caused by 3GC “non-susceptible” strain (*p* = 0.025), received less frequently an active empiric therapy (40.9 vs. 63.6, *p* = 0.015), and had a worse clinical response 7 days after the start of the treatment (31.8% vs. 85.6%, *p* < 0.001).

**Table 4:** Patient characteristics stratified according to 30-day mortality

	Patients survived	Patients not survived	*p* value
N of patients = 217	195	22	
*Demographic variables*			
Male, n (%)	111 (56.9)	9 (40.9)	0.85
Age, median years (IQR)	72 (57–80)	79 (76–85)	**0.004**
Charlson comorbidity index, median (IQR)	2 (0–4)	2 (1–5)	0.43
*Comorbidities and risk factors*			
Comorbidities, n (%):			
Diabetes mellitus	53 (27.2)	8 (36.7)	0.36
Heart failure	31 (15.9)	5 (22.7)	0.41
Chronic kidney disease	48 (24.6)	5 (22.7)	0.85
Haemodialysis	8 (4.1)	0 (0)	0.33
COPD	38 (19.5)	5 (22.7)	0.72
Solid cancer	33 (16.9)	2 (9.1)	0.34
Haematological cancer	6 (3.1)	1 (4.5)	0.71
Neutropenia	0 (0)	0 (0)	/
Chronic hepatopathy	15 (7.7)	2 (9.1)	0.82
Urinary calculosis	24 (12.3)	3 (13.6)	0.86
Solid organ transplantation	1 (0.5)	1(4.5)	0.19
Benign prostatic hypertrophy	22 (11.3)	3 (13.6)	0.74
Devices, n (%):			
Urinary catheter	34 (17.4)	8 (36.7)	**0.03**
Nephrostomy	7 (3.6)	0 (0)	0.37
Ureteral stent	20 (10.3)	1(4.5)	0.39
Invasive procedures on the urinary tract in the previous 30 days, n (%)			0.15
Surgery	4 (2.1)	0 (0)	
Endoscopy	3 (1.5)	1 (4.5)	
Change of urinary catheter/nephrostomy/stent	18 (9.2)	5 (22.8)	
None	170 (87.2)	16 (72.7)	
Antibiotic therapy in previous 3 months, n (%):	41(20.5)	6 (27.3)	0.33
Current antibiotic therapy, n (%):	21(10.8)	2 (9.1)	0.99
Hospitalisation in the previous 3 months, n (%):	25 (12.8)	3 (13.6)	0.80
Hospitalisation in Long-term facilities in the previous 3 months, n (%):	4 (2.1)	4 (18.2)	**0.004**
*Clinical and microbiological variables*			
Severity of infection, n (%):			**0.003**
Non-sepsis	90 (46.1)	2 (9.1)	
Sepsis	81 (41.5)	14 (63.6)	
Septic shock	24 (12.3)	6 (27.3)	
SOFA score, median (IQR)	1 (0–3)	4 (3–5)	** < 0.001**
Agent, n (%):			0.65
*E. coli*	137 (70.3)	14 (63.6)	
*Klebsiella* spp.	37 (19.0)	6 (27.3)	
Other enterobacterales	21 (10.8)	2 (9.1)	
3CG-resistant pathogens. n (%)	66 (33.8)	13 (59.1)	**0.025**
Empirical antibiotic therapy, n (%)			
Amoxicillin/clavulanate or ampicillin/sulbactam	97 (49.7)	7 (31.8)	0.11
Piperacillin/tazobactam	43 (22.0)	10 (45.4)	** < 0.001**
3rd Generation cephalosporins	8 (4.1)	0 (0)	0.33
Carbapenems	5 (2.6)	0 (0)	0.45
Fluoroquinolones	9 (4.6)	0 (0)	0.30
Aminoglycoside	14 (7.2)	0 (0)	0.19
Trimetoprim/sulfamethoxazole	6 (3.1)	0 (0)	0.40
Iv Fosfomycin	7 (3.6)	0 (0)	0.37
Glycopeptides	2 (1.0)	1(4.5)	0.18
Empirical combination therapy, n (%):	17 (8.72)	3 (13.6)	0.45
Appropriate empirical antibiotic therapy, n (%):	124 (63.6)	9 (40.9)	**0.015**
Hospitalisation, n (%)	45 (23.1)	6 (27.3)	0.17
*Outcome*			
Clinical response at 7 days, n (%):	167 (85.6)	7 (31.8)	** < 0.001**

In a multivariate logistic regression analysis (Table 5), a good 7-day clinical response was the only variable protective for 30-day mortality (OR 0.11, 95% CI 0.04–0.36, *p* < 0.001).

**Table 5:** Multivariate analysis for independent predictors of 30-day mortality

	OR	95% CI		*p* value
		Lower	Upper	
Age > 70 years	1.03	0.99	1.07	0.21
Presence of urinary catheter	2.46	0.76	7.97	0.13
Sepsis/septic shock vs. no sepsis	2.77	0.56	13.9	0.21
3GC-R isolate	0.24	0.63	6.09	0.24
Appropriate empirical antibiotic therapy	0.84	0.24	2.92	0.78
Clinical response at 7 days	0.11	0.04	0.36	** < 0.001**

## Discussion

In our study, including 288 patients with community-acquired UTIs due to *Enterobacterales*, 99 (35.9%) subjects had an infection caused by *Enterobacterales* “non-susceptible” to 3GC; with *E. coli* resistant (“I” or “R”) to 3GC in approximately 30% of cases and *K. pneumoniae* in approximately 48%. These prevalences, even though alarming, are similar to those described in the Report of Antimicrobial Resistance in Campania Region in 2022 [12], where *E. coli* was 3GC-R in 28.8% and *K. pneumoniae* in 46.6% of cases, and to those reported in Italy by the European Centre for Disease Prevention and Control (eCDC) in 2023, *i.e.* 3GC-R of 26.7% among *E. coli* and 55.2% among *K. pneumoniae* isolates [7]. Data in the literature regarding 3GC-resistance in UTIs show a great variability according to the geographical location, and mainly report information on extended-spectrum-beta-lactamase (ESBL)-producing pathogens rather than 3GC-resistance per se. In France, a cross-sectional study [13] performed in 2021 reported a 3.0% (1.4–8.8%) mean prevalence of ESBL-producing (ESBL-P) *E. coli* among 444,281 *E. coli* isolates from community urine samples. A retrospective study conducted in Singapore among 869 patients admitted to ED for UTI reported a 42.8% prevalence of resistance to third generation cephalosporins. Lastly, a multicentre retrospective cohort study [14] including 4,107 patients with febrile UTI admitted to 21 EDs in California, reported a prevalence of UTI caused by 3GC-R *E. coli*, *K. pneumoniae* and/or *Proteus mirabilis* of 12.9%. Besides, the prevalence of UTIs caused by 3GC-R pathogens is alarmingly increasing over time, as reported by an Icelandic observational case–control study [15], where authors described an increasing rate of ESBL-P *E. coli* from 2.6% in 2012 to 7.6% in 2021 (*p* < 0.001) in a cohort of 27,747 patients with UTI, all caused by *E. coli*.

Focusing on susceptibility profiles, in our cohort, 47.2% of *E. coli* strains and 61.2% of *Klebsiella* spp. were non-susceptible to aminopenicillin, a mean of 30.6% and 47.9% strains were non-susceptible to 3GC, and 43.3% and 55.1% were non-susceptible to ciprofloxacin, respectively. Interestingly, *Klebsiella* spp. was more frequently non-susceptible to gentamicin than amikacin (26.5% *versus* 0%); and carbapenem-resistant strains were identified in 20% of cases. Empiric therapy included aminopenicillin in 46.6% of patients and was considered appropriate only in 61.1% of them; the appropriateness rate even decreased to 47.3% in case of UTI caused by 3GC-R pathogens. According to our epidemiological data, it is easy to understand why the adequate antibiotic rate was so low. These data are deemed important for the implementation of local guidelines for empiric therapy in the ED. As a matter of fact, starting from these results, we should change the habits of prescribing beta-lactams monotherapy as first line treatment in critically ill patients admitted to ED with clinical suspicion of UTI. A potential alternative is represented by combination regimens including an aminoglycoside, as suggested by several preclinical data showing a synergistic activity between aminoglycosides and beta-lactams against multi-drug resistant *Enterobacterales* strains [16, 17].

In our study, patients with infection caused by 3GC-R strains were older, had more comorbidities, more frequently a nephrostomy, a urinary device replacement in the previous 30 days, an history of previous hospitalisation and of long-term care facilities stay. However, the only variable that was independently associated with the isolation of a 3GC-R pathogen was the admission to a hospital in the previous three months. These results are in line with the literature, even if, as stated above, studies mainly focus on factors associated with ESBL production rather than with 3GC-resistance [15, 18, 19]. A systematic review [18] published in 2020, including 16 observational studies and 12,138 patients, identified male gender, older age, previous antibiotic therapy and/or hospitalisation, and a history or recurrent UTI as main risk factors for community acquired UTI due to ESBL-P *E. coli*. A retrospective study [20] performed in a Chinese tertiary care hospital reported male gender (OR 1.607; 95% CI 1.066–2.416), age (OR 4.1; 95% CI 1.678–12.343), urological procedures (OR 1.81; 95% CI 1.197–2.729), a hospital stay in the previous three months (OR 1.872; 95% CI 1.141–3.067) and antibiotic use in the previous three months (OR 1.833; 95% CI 1.055–3.188) as factors independently associated with ESBL-P *Enterobacterales* (ESBL-E) infection among 874 patients with UTI. Similar data were described by another retrospective case–control study performed in the US in a cohort of 251 adults with ESBL-E UTI matched 1:1 with 251 controls with a non-ESBL-E UTI. In univariate analysis, history of recurrent UTIs, neurogenic bladder, presence of urinary catheter, and exposure to 3GC or fluoroquinolones in the previous three months were associated with higher risk of ESBL UTIs; at the multivariate analysis, a history of repeated UTIs (adjusted OR, aOR, 6.40; 95% CI 3.42–12.66) and prior antibiotic exposure (aOR, 7.98; 95% CI 2.92–28.19) remained independently associated with ESBL infection [19]. Furthermore, several anthropogenic factors, such as primary care, hospitals and animal farming, should be considered as risk factors of ESBL-*E.coli* in the community setting, as reported by Larremendy et al. in 2021 [21].

Regarding the prognosis *quoad vitam*, in our study patients with an infection due to 3GC-R *Enterobacterales* showed a more severe infection, experienced a lower 7-day clinical response and had a higher 30-day mortality. Moreover, patients who did not survive at 30 days were older, were more frequently admitted to a long-term facility, experienced more severe infections, had a higher rate of infections due to strains non-susceptible to 3GC, received less frequently an active empiric therapy, and had a worse clinical response 7 days after the start of the treatment. However, a good clinical response at 7 days resulted the only variable protective for 30-day mortality at multivariate logistic regression analysis. In the above mentioned study in 21 EDs in California [14], patients with UTI caused by 3GC-R *E. coli*, *K. pneumoniae* and/or *Proteus mirabilis* had a significant longer hospital-length of stay (adjusted mean difference 29.7 h; 95% CI 19.0–40.4), a higher 90-day mortality (aOR 1.56; 95% CI 1.07–2.28) and a higher risk of an inappropriate empiric therapy (OR 21.95, 95% CI 16.9–26.0) compared to the control group.

Our study has some limitations, *i.e*., we did not have information about bacteraemic infections, nor about the exact timing of initiation of an active and targeted therapy; moreover, results might have a bias since we considered “I” and “R” strains as non-susceptible, and we did not distinguish 3GC-R due to ESBL or AmpC production, since molecular characterization of isolates was not performed. For the same reason, we cannot exclude that some strains testing susceptible to 3GR harboured a repressed AmpC gene. Furthermore, only 22 deaths occurred in our population, and the multivariate analysis is underpowered for this outcome. Lastly, some data may have been missed because of the setting of the study, the ED, where colleagues are often overwhelmed by work, and often patients are discharged home. However, this is a prospective and multicentre study and provides useful data on the prevalence of UTIs caused by pathogens non-susceptible to 3GC and on the epidemiology of the main bacterial strains causing UTIs circulating in these areas, information essential to set up an active empiric therapy.

Further prospective studies are needed to investigate the rate of antimicrobial resistance and guide the implementation of empiric treatment guidelines in high prevalence settings.

## Conclusions

In our study, 35.9% of pathogens isolated in urine cultures of patients with community acquired UTIs were non-susceptible to 3GC; these isolates were associated with an history of admission to a hospital or a long-term care facility in the previous three months.

In the emergency department, the knowledge of local epidemiology and of risk factors for antimicrobial resistance is of paramount importance for choosing the right empiric therapy and setting up local guidelines.

## Supplementary Information

Below is the link to the electronic supplementary material.

Supplementary file1 (DOCX 25 KB)

## Data Availability

The data presented in this study are available on request from the corresponding author.

## Abbreviations

UTI: Urinary tract infection
ED: Emergency department
AMR: Antimicrobial resistance
MDR: Multidrug resistant
3GC: 3rd generation cephalosporin
3GC-R: 3rd generation cephalosporin-resistance
WHO: World Health Organization
eCDC: European centre for disease prevention and control
CRF: Case report form
CCI: Charlson comorbidity index
SD: Standard deviation
IQR: Interquartile range
OR: Odds ratio
aOR: Adjusted OR
CI: Confidence interval
P/T: Piperacillin/tazobactam
TMP/SMX: Trimethoprim/sulfamethoxazole
COPD: Chronic obstructive pulmonary disease
ESBL: Extended-spectrum-beta-lactamase
ESBL-P: ESBL-producing
ESBL-E: ESBL-*Enterobacterales*
LoS: Length of stay
