# Supplementary material for: Prevalence and outcomes of Urinary tract infections caused by Enterobacterales resistant to third-generation cephalosporins in the Emergency Department: results from UTILY cohort, a prospective multicentre study
Source: Infection. 2025 May 9;53(5):2061–72. doi: 10.1007/s15010-025-02547-3 (PMC12460482; doi:10.1007/s15010-025-02547-3)
Supplement: Supplementary file 1 — Supplementary file1 (DOCX 25 KB) [file 15010_2025_2547_MOESM1_ESM.docx]

**Supplementary Table 1** Demographic, clinical and microbiological characteristics of patients included in the study according to urban areas

|  | **Naples**  **N=155** | **Caserta**  **N=119** |
| --- | --- | --- |
| **DEMOGRAPHIC VARIABLES** |  |  |
| Male, n (%) | 70 (45.2) | 45 (37.8) |
| Age, median years (IQR) | 72 (59; 80) | 70 (51;80) |
| Charlson comorbidity index, median (IQR) | 2 (0;4) | 0 (0;2) |
| Devices, n (%):   - Urinary catheter - Nephrostomy - Ureteral stent | 22 (14.2)  6 (3.9)  21(13.5) | 27 (22.7)  1 (0.8)  1 (0.8) |
| Invasive procedures on the urinary tract in the previous 30 days, n (%)   - Surgery - Endoscopy - Change of urinary catheter/nephrostomy/stent | 3 (1.9)  3 (1.9)  18 (11.6) | 1 (0.8)  4 (2.7)  8 (6.7) |
| Antibiotic therapy in previous 3 months, n (%): | 42 (27.1) | 19 (15.9) |
| Current antibiotic therapy, n (%): | 19 (12.3) | 13 (10.9) |
| Hospitalisation in the previous 3 months, n (%): | 19 (12.3) | 12 (10.1) |
| Hospitalisation in Long-term facilities in the previous 3 months, n (%): | 4 (2.6) | 6 (5.0) |
| **CLINICAL AND MICROBIOLOGICAL VARIABLES** |  |  |
| Severity of infection, n (%):   - Non-sepsis - Sepsis - Septic shock | 51 (32.9)  83 (53.5)  20 (12.9) | 74 (62.2)  34 (28.6)  9 (7.6) |
| SOFA score, median (IQR) | 2 (1;4) | 0 (0;3) |
| Agent, n (%):   - *E. coli* - *Klebsiella* spp*.* - Other *Enterobacterales* | 114 (73.6)  25 (16.1)  16 (10.3) | 79 (66.4)  24 (20.2)  16 (13.4) |
| **SUSCEPTIBILITY PROFILE** |  |  |
| 3CG non-susceptible pathogens, n (%) | 59 (38.1) | 38 (31.9) |
| ***E. coli* R/I, n (%)**   - cefotaxime/ceftriaxone - ceftazidime - protected aminopenicillin - piperacillin/tazobactam - meropenem   ***Klebsiella* spp*.* R/I, n (%)**   - cefotaxime/ceftriaxone - ceftazidime - protected aminopenicillin - piperacillin/tazobactam - meropenem   **Other *Enterobacterales* R/I, n (%)**   - cefotaxime/ceftriaxone - ceftazidime - protected aminopenicillin - piperacillin/tazobactam - meropenem | **114 (73.6)**  35 (22.6)  38 (24.5)  57 (36.8)  14 (9.0)  1 (0.6)  **25 (16.1)**  12 (7.7)  12 (7.7)  14 (9.0)  14 (9.0)  5 (3.2)  **16 (10.3)**  4 (2.6)  6 (3.9)  6 (3.9)  0 (0)  0 (0) | **79 (66.4)**  21 (17.6)  22 (18.5)  31 (26.1)  8 (6.7)  0 (0)  **24 (20.2)**  11 (9.2)  11 (9.2)  15 (12.6)  13 (10.9)  4 (2.7)  **16 (13.4)**  3 (2.5)  3 (2.5)  8 (6.7)  2 (1.7)  1 (0.8) |
| Empirical combination therapy, n (%): | 16 (10.3) | 8 (6.7) |
| Hospitalisation, n (%): | 55/91 (60.4) | 13/36 (36.1) |
| **OUTCOME** |  |  |
| Clinical response at 7 days, n (%):  Mortality, n (%)   - at 7 days - at 30 days | 110/135 (81.5)  9/132 (6.8)  14/129 (10.8) | 66/82 (80.5)  3/86 (3.5)  8/83 (9.6) |

**Supplementary Table 2** Antibiotic susceptibility profiles of the main pathogens isolated

| **Pathogen** | **Antibiotic** | **N° of isolates tested (%)** | **N° of isolates I/R (%)** |
| --- | --- | --- | --- |
| *E. coli* (N=203) | - protected aminopenicillin - piperacillin/tazobactam - 3^rd^ generation cephalosporins - cefotaxime (o ceftriaxone) - ceftazidime - meropenem - ciprofloxacin - amikacin - gentamicin - cotrimoxazole - fosfomycin - nitrofurantoin | 193 (95.1)  194 (95.6)  189 (93.1)  193 (95.1)  191(94.1)  194 (95.6)  145 (71.4)  194 (95.6)  167 (82.3)  158 (77.8)  120 (59.1) | 91 (47.2)  24 (12.4)  57 (30.2)  60 (31.1)  1 (0.5)  84 (43.3)  3 (2.1)  26 (13.4)  57 (34.1)  21 (13.3)  2 (1.7) |
| *Klebsiella* spp. (N=53) | - protected aminopenicillin - piperacillin/tazobactam - 3^rd^ generation cephalosporins - cefotaxime (o ceftriaxone) - ceftazidime - meropenem - ciprofloxacin - amikacin - gentamicin - cotrimoxazole - fosfomycin - nitrofurantoin | 49 (92.5)  49 (92.5)  49 (92.5)  49 (92.5)  50 (94.3)  49 (92.5)  43 (81.1)  49 (92.5)  48 (90.6)  32 (60.4)  18 (33.9) | 30 (61.2)  27 (55.1)  24 (49.0)  23 (46.9)  10 (20.0)  27 (55.1)  0 (0)  13 (26.5)  12 (25.0)  9 (28.1)  0 (0) |
| Other *Enterobacterales* (N=32) | - protected aminopenicillin - piperacillin/tazobactam - 3^rd^ generation cephalosporins - cefotaxime (o ceftriaxone) - ceftazidime - meropenem - ciprofloxacin - amikacin - gentamicin - cotrimoxazole - fosfomycin - nitrofurantoin | 31 (96.8)  32 (100.0)  31 (96.8)  31 (96.8)  32 (100.0)  31 (96.8)  25 (78.1)  32 (100.0)  32 (100.0)  21 (65.6)  8 (25.0) | 14 (45.2)  2 (6.3)  7 (22.6)  19 (61.3)  1 (3.1)  12 (38.7)  0 (0)  8 (25.0)  8 (25.0)  8 (33.3)  0 (0) |
